# Sensitivity to the MEK inhibitor E6201 in melanoma cells is associated with mutant *BRAF* and wildtype *PTEN* status

**DOI:** 10.1186/1476-4598-11-75

**Published:** 2012-10-05

**Authors:** Sara A Byron, David C Loch, Candice L Wellens, Andreas Wortmann, Jiayi Wu, John Wang, Kenichi Nomoto, Pamela M Pollock

**Affiliations:** 1 Cancer and Cell Biology Division, Translational Genomics Research Institute, Phoenix, AZ, USA; 2 Institute of Health and Biomedical Innovation, Queensland University of Technology, 60 Musk Avenue, Kelvin Grove, Queensland, 4059, Australia; 3 Eisai Inc., Andover, MA, USA; 4 H3 Biomedicine Inc., Cambridge, MA, USA

**Keywords:** Melanoma, *BRAF*, *PTEN*, MEK inhibition, E6201, PI3K, MAPK

## Abstract

**Background:**

Melanoma is the most lethal form of skin cancer, but recent advances in molecularly targeted agents against the Ras/Raf/MAPK pathway demonstrate promise as effective therapies. Despite these advances, resistance remains an issue, as illustrated recently by the clinical experience with vemurafenib. Such acquired resistance appears to be the result of parallel pathway activation, such as PI3K, to overcome single-agent inhibition. In this report, we describe the cytotoxicity and anti-tumour activity of the novel MEK inhibitor, E6201, in a broad panel of melanoma cell lines (n = 31) of known mutational profile *in vitro* and *in vivo*. We further test the effectiveness of combining E6201 with an inhibitor of PI3K (LY294002) in overcoming resistance in these cell lines.

**Results:**

The majority of melanoma cell lines were either sensitive (IC50 < 500 nM, 24/31) or hypersensitive (IC50 < 100 nM, 18/31) to E6201. This sensitivity correlated with wildtype *PTEN* and mutant *BRAF* status, whereas mutant *RAS* and PI3K pathway activation were associated with resistance. Although MEK inhibitors predominantly exert a cytostatic effect, E6201 elicited a potent cytocidal effect on most of the sensitive lines studied, as evidenced by Annexin positivity and cell death ELISA. Conversely, E6201 did not induce cell death in the two resistant melanoma cell lines tested. E6201 inhibited xenograft tumour growth in all four melanoma cell lines studied to varying degrees, but a more pronounced anti-tumour effect was observed for cell lines that previously demonstrated a cytocidal response *in vitro*. *In vitro* combination studies of E6201 and LY294002 showed synergism in all six melanoma cell lines tested, as defined by a mean combination index < 1.

**Conclusions:**

Our data demonstrate that E6201 elicits a predominantly cytocidal effect *in vitro* and *in vivo* in melanoma cells of diverse mutational background. Resistance to E6201 was associated with disruption of *PTEN* and activation of downstream PI3K signalling. In keeping with these data we demonstrate that co-inhibition of MAPK and PI3K is effective in overcoming resistance inherent in melanoma.

## Background

Melanoma is the most lethal form of skin cancer. The prognosis for patients with metastatic disease is poor, with a median survival of 4–6 months and 5-year survival of 16% for patients with distant metastases [1,2]. This, together with the escalating incidence of melanoma around the world [2,3], highlights the urgent clinical need for the elucidation of effective pharmacologic and biologic agents to approach melanoma treatment.

Almost all melanomas harbour mutations in the Ras/Raf/mitogen-activated protein kinase (MAPK) pathway [4,5]. As such, pharmacologic inhibitors of this pathway constitute a promising approach to the treatment of melanoma. This was demonstrated recently by the specific inhibitor of mutated BRAF, vemurafenib (PLX-4032), which produced a dramatic response in patients with *BRAF*-mutant metastatic melanoma, albeit tempered by the rapid emergence of resistance [6]. Unfortunately, specific targeting of the oncogenic kinase does not guarantee long term clinical success and this study and others [7-9] highlight the plasticity of oncogenic signalling in melanoma cells to overcome drug sensitivity.

It has been proposed that melanomas demonstrate oncogenic addiction to the Ras/Raf/MAPK pathway. With selective BRAF inhibition, melanoma cells can undergo a “kinase switch” allowing the addicted cells to maintain high MAPK signalling and continued malignancy even in the presence of inhibitor [7-9]. For example, Villanueva and associates [9] demonstrated switching to ARAF and CRAF mediated extracellular signal-regulated kinase (ERK)1/2 activation, and upregulation of insulin-like growth factor 1 receptor (IGF-1R)/phosphoinositide 3-kinase (PI3K) survival signalling with chronic BRAF inhibition in melanoma cells. Consistent with these *in vitro* results, they also observed high IGF-1R and phosphorylated Akt in post-relapse tumour biopsies from patients whose metastatic melanoma developed resistance to BRAF inhibition. These findings underscore the importance of not only MAPK signalling but also parallel signalling cascades, like PI3K/Akt/mammalian target of rapamycin (mTOR), in melanoma survival and progression and, as such, the presumed power of combinatorial pathway inhibition.

Pharmacologic inhibitors of mitogen-activated protein kinase/extracellular signal-regulated kinase kinase (MEK1/2) show clear anti-tumour activity in preventing melanoma cell line growth and survival *in vitro*[10-14] and *in vivo*[13,15]. Despite this, they demonstrate little or no improvement over traditional chemotherapy in a clinical setting, although it should be noted that these patients were not pre-screened for specific mutations [16-18]. Interestingly, subanalysis of results from phase II trials in melanoma [17,18] have hinted at a greater efficacy of MEK1/2 inhibition in *BRAF*-mutant patients albeit in small patient numbers (15 and 67 *BRAF* mutant patients). As such, the clinical outcome of future MEK1/2 trials may be improved by identifying markers like *BRAF* to enrich the study with patients more likely to respond [19]. As Ras is thought to provide resistance to BRAF and MEK inhibitors by activation of additional downstream pathways, MEK inhibitors might be best utilised in combination. Interestingly, combined BRAF (GSK2118436) and MEK (GSK1120212) inhibition was recently shown to overcome NRAS-mediated resistance to BRAF inhibition in melanoma cells already harbouring *BRAF*^*V600*^ mutations [20]. The combination therapy potently abrogated ERK signalling, inhibited cell growth and upregulated markers of apoptosis [20]. Furthermore, this drug combination was recently shown to induce tumour regression or stable disease in roughly two-thirds of *BRAF*^*V600*^ mutant melanoma patients refractory to single-agent BRAF inhibition [21]. As such, sequential targeting of the MAPK pathway at multiple nodes in *BRAF* mutant patients (irrespective of their *RAS* mutational status) or targeting of parallel pathways, such as PI3K, in *RAS* mutant patients, may also improve the therapeutic response of melanoma patients to MEK1/2 inhibition [20,22,23].

The aim of the current study was to utilize a diverse melanoma cell line panel (n = 31) of known mutational status (*BRAF*, *HRAS*, *NRAS*, and *phosphatase and tensin homolog* (*PTEN*)) to aid in the identification of a patient population most likely to respond to MEK inhibition. We utilized E6201, a potent, novel inhibitor of MEK1 and MEK kinase-1 [24,25] currently under development as an anti-cancer agent. E6201 is in a Phase I clinical trial for advanced solid malignancies that had an expansion phase to specifically include patients with *BRAF* mutant tumours (including brain metastases) (NCT00794781, ClinicalTrials.gov), and outcome analysis is currently maturing.

## Results

### Sensitivity to E6201 in a melanoma cell line panel

Sensitivity to E6201 was assessed in a panel of 31 cell lines for which the mutation status of common melanoma genes was known (Table 1). These lines were chosen to represent different mutational profiles from a larger panel of more than one hundred melanoma cell lines. Western blots in Additional file 1: Figure S1 confirm that E6201 efficiently inhibits MEK1/2 activity by virtue of its ability to abrogate phosphorylation of ERK1/2 in our entire panel of melanoma cell lines.

**Table 1:** Mutational Analysis of a Melanoma Cell Line Panel

**Cell Lines**	***BRAF***	***NRAS***	***KRAS***	***HRAS***	***PTEN***	***CDKN2A***		***CDK4***	***TP53***
						**p16INK4A**	**p14ARF**		
D35	wt	wt	wt	wt	wt	c.151-1 G > A	wt	nd	wt
UACC1118	wt	wt	wt	wt	wt	wt	wt	wt	wt
UACC2837	wt	wt	wt	wt	wt	wt	wt	wt	p.R342X
MM329	wt	wt	wt	wt	wt	wt	wt	wt	wt
UACC2534	wt	wt	wt	wt	wt	Deletion*	wt	wt	wt
UACC1097	wt	p.Q61K	wt	wt	p.I50N	p.H83Y	p.A97V	wt	wt
M92-001	wt	p.G13R	wt	wt	wt	Deletion*	Deletion*	wt	wt
UACC3074	wt	p.G13R	wt	wt	wt	p.P114L	wt	wt	Deletion
UACC3093	wt	p.Q61L	wt	wt	wt	wt	wt	wt	wt
UACC383	wt	wt	wt	p.Q61R	wt	Deletion*	Deletion*	wt	wt
UACC647	p.V600E	wt	wt	wt	Deletion*	wt	wt	wt	Deletion
UACC558	p.V600E	wt	wt	wt	c.802-1 G > C	wt	wt	wt	wt
MM200	p.V600E	wt	wt	wt	p.F56I	wt	wt	wt	wt
UACC903	p.V600E	wt	wt	wt	p.Y76X	Deletion*	Deletion*	wt	wt
BL	p.V600E	wt	wt	wt	p.Q298X	p.E88K	p.G102E	wt	p.I195T
MM622	p.V600E	wt	wt	wt	p.L139X	p.G67fs*53	p.R122fs*79	wt	wt
NK14	p.V600E	wt	wt	wt	p.P38L	p.R58X	p.P72L	wt	wt
M91-054	p.V600E	wt	wt	wt	wt	wt	wt	wt	wt
D17	p.V600E	wt	wt	wt	wt	wt	wt	wt	wt
MM604	p.V600E	wt	wt	wt	wt	wt	wt	wt	wt
MM170	p.V600E	wt	wt	wt	wt	Deletion*	Deletion*	wt	wt
UACC091	p.V600R	wt	wt	wt	wt	Deletion*	Deletion*	wt	p.R213X
MM229	p.L597S	wt	wt	wt	wt	Deletion*	Deletion*	wt	wt
WSB	p.V600E	wt	wt	wt	wt	Deletion*	Deletion*	wt	wt
A375	p.V600E	wt	wt	wt	wt	p.E61X; p.E69X	c.1_316del316; p.G75V; p.G83V	wt	wt
MM540	p.V600E	wt	wt	wt	wt	wt	wt	wt	p.S366P
SKMEL13	p.V600E	wt	wt	wt	wt	Deletion*	Deletion*	wt	p.R248W
UACC257	p.V600E	wt	wt	wt	wt	p.P81L	wt	wt	wt
WM35	p.V600E	wt	wt	wt	wt	Deletion*	Deletion*	wt	wt
UACC1022	p.L597S	wt	wt	wt	wt	wt	wt	wt	p.R196X
UACC1308	p.V600E	wt	wt	wt	wt	wt	wt	wt	p.F134S

wt = wildtype, * = homozygous, nd = not determined.

**Figure 1:**
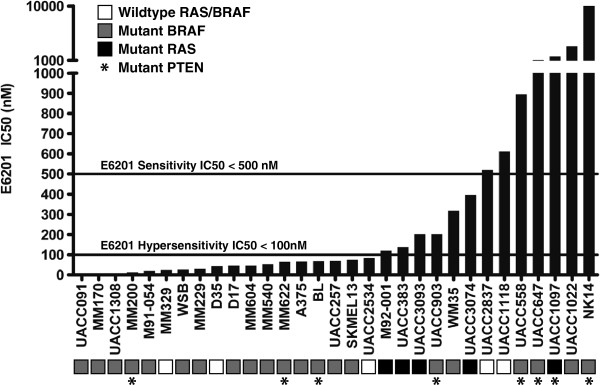
** Sensitivity of a melanoma cell line panel to E6201.** The *in vitro* cell viability for all 31 cell lines studied was determined by MTS or Sulforhodamine-B (SRB) assay (for MM329). IC50 < 500 nM was considered sensitive and IC50 < 100 nM was considered hypersensitive.

The majority (24/31) of the melanoma cell lines were sensitive to E6201 (IC50 <500 nM) (Figure 1). MAPK activation due to mutations in *BRAF* and *NRAS* was not significantly associated with increased sensitivity to E6201. In the 26 cell lines carrying mutations in *BRAF*, *NRAS*, or *HRAS*, sensitivity to E6201 was statistically associated with wildtype *PTEN* status (p = 0.02). Specifically, of the 18 cell lines with wildtype *PTEN*, 17 were sensitive whereas in the 8 cell lines with mutant *PTEN*, only 4 were sensitive. Moreover, even if *PTEN* status alone is examined, E6201 sensitivity is associated, albeit non-significantly, with wildtype *PTEN* status; 23/31 cell lines are wildtype for *PTEN* and of these 20 are sensitive (whereas only 4/8 cell lines with mutant *PTEN* are sensitive) (p = 0.053). Interestingly, 18 of the 24 sensitive cell lines also demonstrated hypersensitivity to E6201, with an IC50 < 100 nM. Using this criterion, *BRAF* mutation status correlated with E6201 hypersensitivity (p < 0.03), with 15 out of the 18 hypersensitive cell lines possessing a *BRAF* mutation. In contrast, of the 11 cell lines with wildtype *BRAF*, only 3 were hypersensitive. In those cell lines carrying mutations in *BRAF* (21 cell lines), sensitivity to E6201 was not statistically associated with wildtype *PTEN* status. *NRAS/HRAS* mutation status correlated with E6201 resistance, where none of the 5 *NRAS*/*HRAS* mutant cell lines were hypersensitive to E6201 and 18 of the 26 *NRAS/HRAS* wildtype cell lines were hypersensitive (p < 0.01). Neither *CDKN2A*, *CDK4* or *TP53* mutational status in our panel of melanoma cell lines, irrespective of their *BRAF* and *RAS* mutational status, was associated with E6201 sensitivity.

### E6201 sensitivity and downstream pathway activation

**Figure 2:**
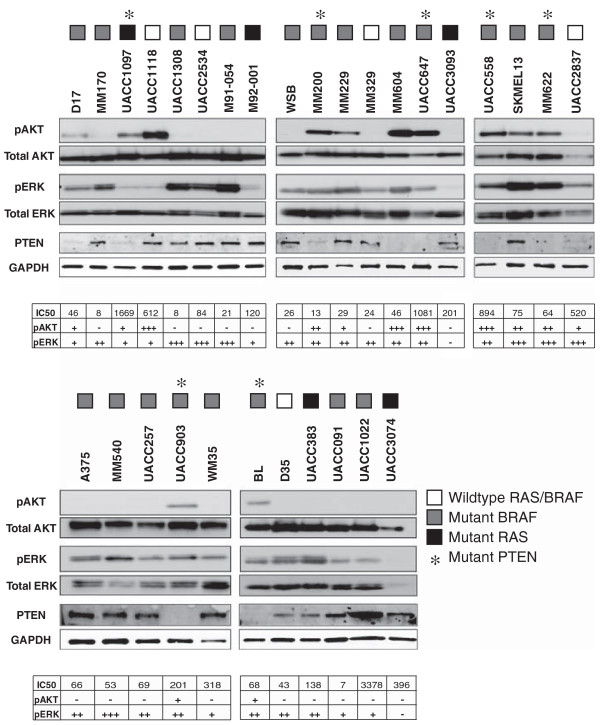
** Downstream PI3K and MAPK pathway activation.** Melanoma cells were starved in 0.2% FBS overnight and then protein lysates were collected and evaluated by Western blot analysis for activation of ERK1/2 and Akt. Numerical values for the IC50 of E6201 for each cell line, as well as phosphorylation status scoring for both ERK1/2 and Akt is provided in tabular form.

To determine whether E6201 responsiveness correlated with direct Akt or ERK1/2 activation, the phosphorylation status of Akt and ERK1/2 proteins was evaluated following serum starvation (Figure 2). Phosphorylated (p) Akt (Ser473) was detectable in 7/7 cell lines with mutant PTEN. In addition, pAkt was present in 5/23 cell lines with wildtype *PTEN* although the mechanism responsible for phosphorylation of Akt in these cell lines is unknown. Phosphorylated (p) ERK1/2 was detected in all cell lines with mutant *BRAF* (20/20). Consistent with previous reports [13,26], elevated pERK1/2 was detected in 3/5 cell lines with mutant *NRAS* or *HRAS*. All five cell lines with wildtype *BRAF* and *NRAS* also had elevated ERK1/2 phosphorylation, as reported previously [26,27], although the mechanism responsible for ERK1/2 activation in these cell lines is unknown. When the cell lines were classified based on phospho-ERK levels rather than *BRAF* mutation status, there was no correlation with the degree of cell growth inhibition. In contrast, high levels of pAkt (3+) in *BRAF/RAS* mutant cell lines were strongly suggestive of insensitivity to E6201 (p = 0.057). Furthermore, high levels of pAkt (3+) significantly correlated with E6201 insensitivity independent of *BRAF* or *PTEN* status (p < 0.02). PTEN protein was present in 20 of the melanoma cell lines tested with a lack of the tumour suppressor being suggestive of resistance to E6201 in not only *BRAF/RAS* mutant lines (p = 0.12) but also if all lines are considered (p = 0.14). 

### Characterization of E6201 response *in vitro*

**Figure 3:**
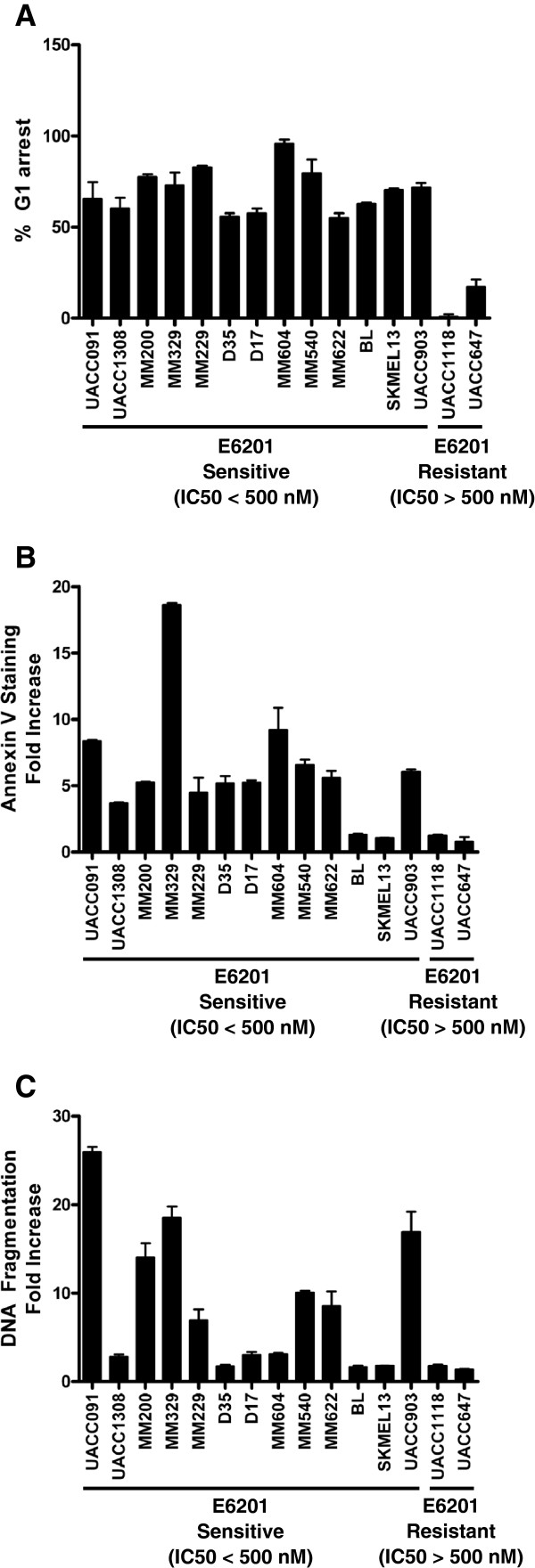
** Characterization of E6201 response in a subset of melanoma cell lines.A**. DNA content as a measure of cell cycle progression with E6201 treatment. A subset of sensitive and resistant melanoma cell lines were treated with 200 nM E6201 for 48 hours, after which cell cycle analysis was performed by propidium iodide staining and flow cytometry. The percent increase in cells in G1 phase with E6201 therapy is shown for 13 sensitive and 2 resistant melanoma cell lines. E6201 treatment resulted in an accumulation of cells in G1 phase of the cell cycle in all sensitive melanoma lines studied. No such accumulation was noted in E6201-resistant melanoma lines **B**. Cell death as assessed by Annexin V positivity after treatment of E6201. After 72 hours of 200 nM E6201 treatment, melanoma cells were analysed for Annexin V-FITC–positive cells by flow cytometry. MEK inhibition by E6201 resulted in a greater than 2-fold increase in Annexin V–positive cells, indicative of apoptosis, in most (11 out of 13) sensitive melanoma lines. No such increase was observed in 2 melanoma cell lines previously shown to be resistant to E6201. **C**. Determination of DNA fragmentation after E6201-induced cell death. After 72 hours of 200 nM E6201 treatment, a Cell Death Detection ELISA (Roche) was performed as per the manufacturer’s instructions. Treatment with E6201 resulted in increased DNA fragmentation (greater than 2-fold) in 10 out of 13 sensitive melanoma lines and did not induce significant DNA fragmentation in any of the E6201-resistant melanoma cell lines.

MEK inhibitors have been previously shown to have a predominantly cytostatic effect on melanoma cells, although some clinically relevant inhibitors, such as CI-1040, PD0325901 and AZD6244, have been shown to induce cell death [10,12,13]. We sought to further evaluate the mechanism of sensitivity to E6201, as an equivocal cytocidal response *in vitro* may equate to the poor clinical response observed with current MEK inhibitors. Fifteen melanoma cell lines were selected such that 13 cell lines demonstrated sensitivity to E6201 and 2 cell lines were insensitive to E6201. Of these cell lines, seven were mutant for *BRAF* but wildtype for *PTEN*, five were mutant for both *BRAF*/*NRAS* and *PTEN*, and three were wildtype for both *BRAF* and *PTEN*. E6201 treatment induced G1 arrest in all of the sensitive cell lines and had little to no effect on cell cycle progression in the two insensitive cell lines (Figure 3A). E6201 treatment resulted in a greater than 2-fold increase in Annexin-positive staining in eleven out of fifteen cell lines, including eleven out of thirteen sensitive cell lines (Figure 3B). Two sensitive cell lines, SKMEL13 and BL, did not demonstrate E6201-induced Annexin staining although both of these cell lines underwent cell cycle arrest with E6201 treatment and were hypersensitive to E6201 (IC50 < 100 nM). These experiments were repeated in duplicate to confirm this finding. E6201 induced a less than two-fold increase in Annexin staining in the E6201-insensitive cell lines (IC50 > 500 nM) (Figure 3B). Three of the five *PTEN*-mutant cell lines exhibited a cytocidal response to E6201, demonstrating that *PTEN* mutation does not preclude a cytocidal response to E6201. E6201 also induced cell cycle arrest and cell death in cell lines with constitutively active Akt, suggesting that although high pAkt correlates with E6201 insensitivity, cell lines with high pAkt can still undergo a cytocidal response to E6201. 

To confirm our Annexin V results we also performed an enzyme-linked immunosorbent assay (ELISA) to determine the degree of DNA fragmentation as an indicator of cell death with E6201 treatment (Figure 3C). The results from the cell death ELISA were very similar to that obtained from the Annexin studies with 10 out of 13 sensitive melanoma lines demonstrating a greater than two-fold increase in DNA fragmentation with E6201. Of the three sensitive lines that did not exhibit a cytocidal response by ELISA, SKMEL13 and BL also demonstrated no induction of cell death with E6201 by Annexin positivity, as stated previously. There was no significant induction of DNA fragmentation in any of the E6201-resistant melanoma cell lines.

### Characterization of E6201 response *in vivo* in melanoma xenografts

**Figure 4:**
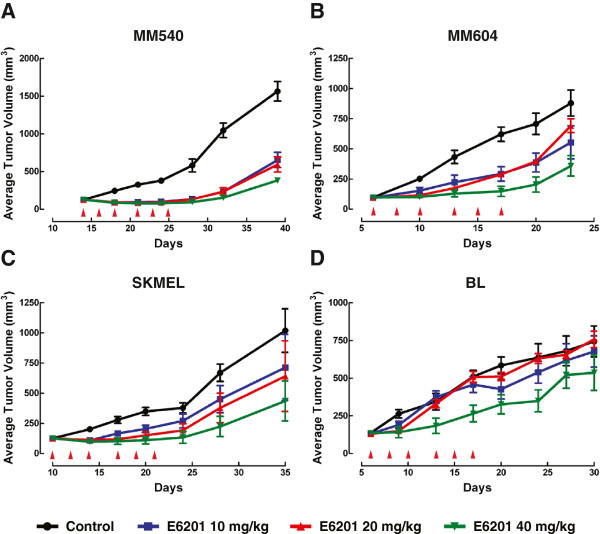
** Anti-tumour effects of E6201 on melanoma xenografts.** Growth curves of MM540 (**A**), MM604 (**B**), SKMEL13 (**C**) and BL (**D**) melanoma xenografts from mice treated with vehicle control and E6201 (10, 20 and 40 mg/kg). Red arrows indicate the days at which mice were treated with vehicle or E6201.

We evaluated the *in vivo* activity of E6201 in two melanoma cell lines that exhibited a cytocidal response (MM540, MM604) and two melanoma cell lines that exhibited a cytostatic response (SKMEL13, BL) to E6201 *in vitro* (Figure 4). Given that the majority of sensitive melanoma cell lines in our cell line panel exhibited a cytocidal response to E6201 *in vitro*, we hypothesized that E6201 would induce tumour regression in a xenograft model of these cell lines as well, and to a greater extent in those cell lines that demonstrated a cytocidal response to E6201 *in vitro* compared to those with a cytostatic response.

Administration of E6201 at all doses (10, 20 and 40 mg/kg) to MM540 tumour-bearing mice completely abrogated tumour growth and caused transient, partial tumour regression for the two weeks of drug treatment, although tumour growth recommenced following drug withdrawal, indicating not all cells were killed in this two week period (Figure 4A). E6201 at 40 mg/kg in MM604 and SKMEL13 xenografts prevented tumour progression for the two weeks of drug treatment, with tumour growth recommencing following drug removal, while lower doses of drug (10 and 20 mg/kg) only attenuated, rather than prevented, tumour growth *in vivo* (Figure 4B and C). Only the highest dose of E6201 (40 mg/kg) had any significant inhibitory effect on tumour growth in BL tumour-bearing mice, while lower drug doses had little or no effect on tumour progression (Figure 4D). As such our hypothesis was confirmed, with E6201 inhibiting xenograft tumour growth in all four melanoma cell lines studied, and enhanced *in vivo* activity observed for those cell lines that demonstrated a cytocidal response *in vitro*.

### E6201 and LY294002

Given our previous data suggesting that E6201 resistance is associated with mutation of *PTEN* and high levels of pAkt, we hypothesized that combining E6201 with an inhibitor of the PI3K pathway in these cell lines might result in either an additive or synergistic effect. Additional file 2: Figure S2 demonstrates that LY294002 effectively inhibits PI3K by evidence of reduced phosphorylated AKT protein levels in the four *PTEN*-mutant melanoma cell lines that normally express high levels of pAKT (UACC647, UACC558, UACC903 and MM622). In addition, Additional file 3: Figures S3 and Additional file 4: Figure S4 show the concentration-effect curves for single-agent LY294002 and E6201 respectively, where both drugs were added 24 hours following plating. The six melanoma cell lines tested displayed similar trends in E6201 sensitivity compared to our previous experiments, with MM622, MM540, UACC903, and WM35 being the most sensitive (IC50 = 40-61 nM) and UACC558 and UACC647 being less sensitive (302 and 2310 nM, respectively). Surprisingly, all cell lines showed similar sensitivity to LY294002, with IC50 ranging from 11 μM to 17 μM. This was unexpected, as one would predict MM540 and WM35 cells to be relatively resistant to PI3K inhibition given the lack of detectable levels of pAkt indicating no constitutive PI3K activation in these cell lines. A previous study by Smalley and others [26], however, reported a similar sensitivity of WM35 cells to LY294002.

**Figure 5:**
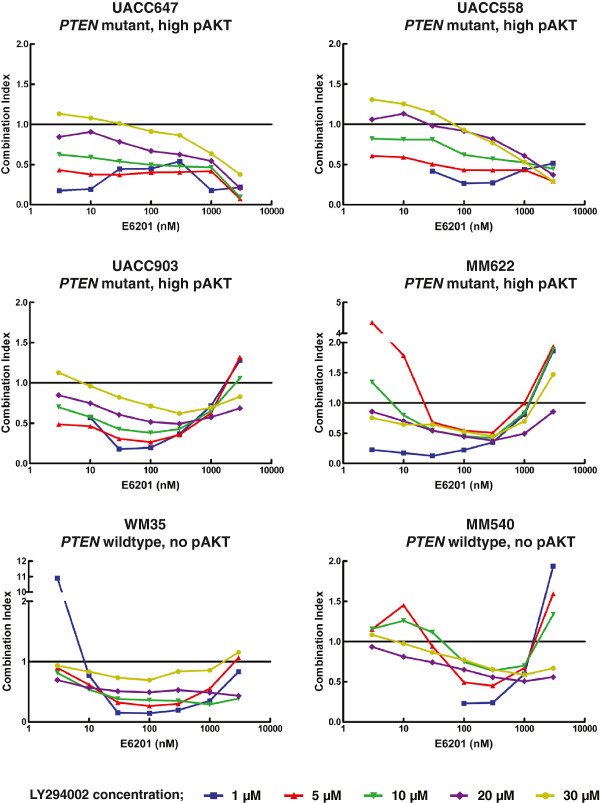
** Combination of E6201 with an inhibitor of PI3K signalling.** Melanoma cells were treated with increasing concentrations of E6201 (3 nM to 3 μM) in combination with 1, 5, 10, 20 and 30 μM LY294002. Synergy with co-inhibition of MEK and PI3K was determined by calculation of a combination index at each concentration. A combination index value of 1 indicates additivity, values < 1 indicate synergism, and values > 1 indicate antagonism. All six of the melanoma cell lines studied showed synergy with the combination of E6201 and LY294002, particularly between an E6201 concentration of 10nM to 1 μM. Interestingly, the more resistant cell lines, UACC647 and UACC558, demonstrated decreasing CI, and hence increasing synergy, with increasing concentrations of E6201.

The concentration response curves for E6201 and LY294002 combinations, normalized to a dimethyl sulfoxide (DMSO) control are given in Additional file 4: Figure S4. As differences in synergy may exist at different drug effect levels, we graphed individual combination index values for LY294002 with increasing concentrations of E6201 for each cell line (Figure 5A). As shown in Figure 5A, evaluating the individual combination index for all combinations tested revealed that E6201 and LY294002 exhibit synergistic activity in all six melanoma cell lines, irrespective of E6201 sensitivity or *PTEN* or pAkt status. Interestingly, different patterns of synergy were observed among the groups of cell lines tested. While most (4/6) of the cell lines showed an increasing combination index (and thus decreasing synergy) at higher concentrations of E6201, UACC647 and UACC558 cells showed a decreasing combination index or enhanced synergy with increasing concentrations of E6201. Notably, this pattern observed for UACC647 and UACC558 cells occurs within the context of high pAkt and relative resistance to E6201, supporting the hypothesis that administration of a PI3K inhibitor can sensitize E6201-resistant cells with high pAkt levels to E6201.

In summary, the combination of E6201 and LY294002 resulted in synergistic activity in all six melanoma cell lines tested, as defined by a combination index < 1. Interestingly, enhanced synergy of E6201 with LY294002 treatment in the E6201-resistant cell lines UACC647 and UACC558 was observed at high concentrations of E6201.

## Discussion

E6201 is a novel MEK1/2 inhibitor which inhibits selected cancer-specific kinases that is currently in clinical trials for solid tumours and, as a result of the data presented herein, is undergoing Phase I expansion in *BRAF*-mutant malignancies (NCT00794781, ClinicalTrials.gov). In the current study, we established a diverse cell line panel to not only represent the known genetic heterogeneity in melanoma, but also to enrich for rare mutations or genotypes in which to test the effectiveness of E6201 *in vitro* and *in vivo*. From this genetically diverse panel, we demonstrate for the first time that sensitivity to MEK1/2 inhibition *in vitro* correlated with wildtype *PTEN* suggesting parallel signalling of the PI3K/Akt/mTOR pathway may play a role in the resistance of melanoma cell lines to E6201 and MEK1/2 inhibitors in general. To this end we demonstrate that concurrent targeting of the Ras/Raf/MAPK and the PI3K/Akt/mTOR pathways was more effective than targeting either of the pathways alone in all six cell lines studied with the greatest synergy observed in E6201 resistant cell lines. These results underscore the power of heterogeneous cell line panels, such as the NCI60, to identify potential biomarkers of sensitivity and resistance in a clinical setting [28].

There is a general consensus that genomic analysis of tumours through The Cancer Genome Atlas (TCGA) and the International Cancer Genome Consortium (ICGC) will identify the core pathways activated in each tumour. Previous work in pancreatic cancer indicates that only 12 pathways need to be activated [29]. This has been interpreted as molecular targeting of only a few pathways may be needed to effectively treat cancer. Emerging *N-Ras/BRAF/ERK* data would suggest that some therapies will only work on pathways activated at a certain “node”. For example, melanoma cells demonstrate marked differences in response to MEK1/2 inhibition, with *BRAF* and *RAS* mutational status thought to predict sensitivity and resistance, respectively. Melanomas harbouring mutant *BRAF* and wildtype *RAS* are intimately dependent on ERK signalling for their growth and survival and selective RAF inhibition in these lines efficiently blocks ERK activation and growth. Conversely, RAF inhibitors paradoxically enhance ERK activation and proliferation in *BRAF*-wildtype, *RAS*-mutant melanoma (and normal) cells through a mechanism that involves the interaction of these drugs with RAF dimers [7,30,31]. In this setting, concurrent treatment with a MEK inhibitor may prevent this paradoxical activation [21,32].

The exquisite sensitivity of *BRAF* mutant cell lines to E6201 is consistent with that reported for other MEK inhibitors, including CI-1040 [13] and AZD6244 (ARRY-142886) [11]. Similar to these MEK inhibitors, *RAS* mutant cell lines do not display the same sensitivity to E6201 as *BRAF* mutant cell lines [11,13]. It is possible that the resistance of *RAS* mutant tumour lines in this study and others is the result of compensatory signalling by a parallel or non-canonical pathway, such as PI3K/Akt/mTOR. Indeed, the importance of intact PI3K signalling has recently been established for Ras-driven lung tumourigenesis *in vivo*[33]. Interestingly, those cell lines with wildtype *BRAF* and *RAS* were not all resistant to E6201 in contrast to previously published data [13], suggesting that these cell lines may carry activation of the MAPK pathway through additional mechanisms, such as receptor tyrosine kinase [34] or MEK1 activation [22]. Perhaps only the combination of genome-wide expression profiling, exome mutation data and phospho-protein status will allow us to unravel these complex pathway interactions and their relative roles in drug sensitivity.

Strangely, despite correlating *BRAF* mutational status to anti-tumour activity with E6201, phosphorylated ERK1/2 levels did not correlate with the magnitude of cell growth inhibition. Similarly, the cytostatic response of melanoma cell lines to other MEK inhibitors has been shown previously not to correlate with pERK levels before or after treatment [26]. Taken together these results support the notion that the upstream mechanism of ERK activation is important in predicting sensitivity to MEK inhibition. These findings also suggest that the cytostasis induced by MEK inhibition may be mediated by modulation of parallel signalling pathways potentially via ERK-mediated autoregulatory processes. To this end, Gopal and co-workers [12] demonstrated reduced efficacy of MEK inhibition in melanoma cell lines as a result of PI3K pathway activation via a MEK-IGF-1R-mediated feedback loop.

Consistent with the role of the MAPK pathway in G1/S transition [35], E6201 exerted cytostatic effects, resulting in G1 arrest *in vitro* and tumour growth inhibition *in vivo*. E6201 also induced cell death in the majority of E6201-sensitive cell lines. It would be interesting to perform a functional genomics screen in those cell lines that only showed growth arrest but not cell death to identify the genes or pathways that could be targeted alongside MEK to induce synthetic lethality. There are previous reports of MEK inhibitors leading to cell death in a subset of sensitive melanoma cell lines. For example, CI-1040 treatment resulted in cell death in 1 out of 4 melanoma cell lines evaluated [13], and cell death in melanoma cell lines has also been reported with its daughter compound, PD0325901 [10]. The MEK inhibitor UO126 has also been reported to lead to caspase-independent cell death in melanoma cell lines [14]. Thus, the cell death we see upon E6201 treatment reflects the potential for MEK inhibition to result in cell death in a specific subset of melanoma cell lines. The cytocidal activity of E6201, however, may also reflect the “multi-target” nature of E6201, such that the cell death observed is due to inhibition of other cancer-specific kinases, such as Src [36]. Indeed, while treatment of melanoma cell lines with the Src inhibitor dasatinib has been shown to inhibit proliferation and invasion [37,38], in some melanoma cell lines it did induce apoptosis [39]. Although clinical responses have been seen in a subset of patients in Phase I and II trials of Dasatinib, biomarkers that predict sensitivity have not yet been identified [40,41]. To validate our findings with E6201 in monolayer culture, we created mouse xenograft models. We hypothesized that E6201 would induce tumour regression in xenografts of sensitive melanoma cell lines, as most of the sensitive melanoma lines in our panel demonstrated cell death (Annexin positivity) in response to E6201 *in vitro*. To this end, we evaluated the *in vivo* activity of E6201 in two melanoma cell lines that exhibited a cytocidal response (MM540, MM604) and two melanoma cell lines that exhibited a cytostatic response (SKMEL13, BL) to E6201 *in vitro*. E6201 dose-dependently inhibited tumour progression in all four of these melanoma xenografts. Furthermore, transient regression was also observed in those cell lines that demonstrated a cytocidal response to E6201 *in vitro*. This is in accordance with previous work showing transient, partial tumour regression in *BRAF* mutant xenografted tumours with MEK1/2 inhibition [13,15]. Furthermore, higher doses of inhibitor were required to limit tumour progression in *BRAF* wildtype and also *NRAS* mutant melanoma xenografts [13].

The cell line panel in this study was selected to include a subset of melanoma cell lines with *PTEN* mutations so that we could evaluate whether *PTEN* mutational status was associated with resistance to E6201. *PTEN* is a tumour suppressor protein and an important negative regulator of PI3K signalling as it inhibits Akt phosphorylation and activation indirectly by hydrolysing the secondary messenger phosphatidylinositol 3,4,5-trisphosphate (PIP_3_) [42]. Indeed, using this cell line panel, we found that insensitivity to E6201 was not only associated with mutant *PTEN* but also high phospho-Akt levels. This finding is consistent with the pro-survival function of Akt signalling [43] and has been observed previously in lung cancer [44] as well as melanoma [12]. Interestingly, two of our resistant cell lines demonstrated no basal PI3K/Akt activation, suggesting an alternative pathway to resistance. It is possible, however, that these resistant cell lines simply activated PI3K/Akt in response to MAPK inhibition, as observed by Gopal *et al*. [12] in melanoma cell lines. Conversely, E6201 induced cell cycle arrest and cell death in some cell lines with constitutively active Akt, suggesting that although high pAkt does correlate with E6201 insensitivity, cell lines with high pAkt (as well as mutant *PTEN*) can still undergo a cytocidal response to E6201. Nonetheless, our findings highlight the possible clinical utility of mutational and oncogenic pathway screening to stratify patients to particular treatments.

PI3K inhibitors have previously been shown to be effective in melanoma cell lines not only in combination with MAPK inhibitors [9,12,45,46], but also in monotherapy [47]. In a mouse model of cutaneous melanoma, Bedogni and colleagues [48] demonstrated that combined targeting of MAPK and PI3K significantly decreased tumour development and incidence more so than either agent given alone. Our findings confirm and expand on this previous work. We show that inhibition of the PI3K pathway in E6201-resistant cell lines with high levels of phosphorylated Akt can sensitize these cell lines to E6201. Indeed, synergy between the PI3K inhibitor, LY294002, and E6201 was evident in all 6 cell lines tested, irrespective of *PTEN* mutation status, pAkt levels, or E6201 sensitivity. Interestingly, the greatest enhancement of E6201 activity by LY294002 occurred in those cell lines that were resistant to E6201 alone. On this note, multiple pharmaceutical companies are testing the effectiveness of combined MEK inhibition and PI3K or AKT inhibition in solid tumours including melanoma. There is also a Phase II trial testing the efficacy of the AZD6244 MEK inhibitor and MK-2206 AKT inhibitor in patients with relapsed BRAF V600E melanoma (NCT01510444, ClinicalTrials.gov).

Recent experience with vemurafenib has demonstrated that personalized cancer therapy can have a significant impact on patient response in this emerging era of molecularly targeted therapy. It is yet to be determined, however, whether MEK inhibitors can also impart meaningful clinical benefits to melanoma patients. To this end, recent preliminary results from a phase I clinical trial of the MEK1/2 inhibitor GSK1120212 in selected solid malignancies with a high frequency of *BRAF* mutation (melanoma, pancreatic, non-small cell lung and colorectal cancers) were impressive with just under three quarters of *BRAF* mutant melanoma patients demonstrating either a partial response or stable disease with therapy [49]. Furthermore, several phase I trials (NCT01271803, NCT01072175 and NCT01231594, ClinicalTrials.gov) are currently assessing dual BRAF and MEK inhibition to target this oncogenic pathway at multiple levels.

## Conclusions

MEK inhibitors are being extensively evaluated in melanoma patients both as single agents and in combination with chemotherapy with thus far equivocal results. From our panel of melanoma cell lines we identified expression of wildtype *PTEN* as a potential genetic marker that may predict sensitivity to MEK1/2 inhibition in melanoma patients. Consistent with this finding, we further implicate involvement of PI3K/Akt/mTOR signalling in modulating sensitivity to MEK1/2 inhibition in melanoma, which is consistent with previous studies [9,12,45,46]. As such, PI3K inhibition may overcome resistance when given in combination with a MEK inhibitor as we have shown here. Our findings confirm the notion that refining patient selection based on the mutational and signalling status of relevant oncogenes and tumour suppressors such as *PTEN* is a powerful clinical tool for the targeted application of emerging agents in melanoma treatment.

## Methods

### Drugs

LY294002 (PI3K inhibitor) was purchased from Calbiochem (La Jolla, CA). E6201 was a kind gift from Eisai Inc. (Andover, MA). E6201 and LY294002 stock solutions were all dissolved in DMSO and used at the concentrations described.

### Cell lines

The melanoma cell lines used in this study and their mutational status are listed in Table 1. This panel was chosen from a larger cohort of well characterized melanoma cell lines to enrich for common and “rare” mutation genotypes, such as joint *BRAF* and *RAS* wildtype status and wildtype *PTEN* status, in order to increase the likelihood of detecting significant associations. Cells were grown in DMEM plus 10% foetal calf serum.

Melanoma cell lines prefixed with “MM”, as well as BL, NK14, WSB, A375 and SKMEL13, were kindly provided by Dr Nick Hayward of the Queensland Institute of Medical Research, Brisbane, Australia. Those cell lines prefixed with “UACC” were originally obtained from the Arizona Cancer Center Tissue Culture Shared Resource, University of Arizona, Tucson, USA and were kindly provided by Dr Jeffrey Trent (National Human Genome Research Institute, NIH, Bethesda, USA) along with the WM35, M91-054 and M92-001 cell lines. We would also like to thank the Australasian Biospecimen Network and Chris Schmidt (Queensland Institute of Medical Research, Brisbane, Australia) for the D17 and D35 cell lines.

### Mutational analysis

Mutational analysis was “generally” performed as previously reported using Sanger sequencing. Sequencing primers for each gene were as previously reported; *BRAF*[50], *NRAS*[50], *KRAS*[51], *PTEN*[51], *CDKN2A*[52] and *TP53*[53]. Those primers used to sequence *HRAS* and *CDK4* in this study are available on request.

The accession numbers for the protein and coding DNA sequences used in our mutational analysis were taken from GenBank and are as follows: *BRAF*;  NM_004333.4   and  NP_004324.2,   *NRAS*;   NM_002524.4 and  NP_002515.1, *KRAS*; NM_004985.3 and NP_004976.2, *HRAS*; NM_005343.2 and NP_005334.1, *CDKN2A     p16INK4A*;  NM_000077.4   and  NP_000068.1, *CDKN2A p14ARF*; NM_058195.3 and NP_478102.2, *CDK4*; NM_000075.3 and NP_000066.1 and *TP53*; NM_000546.5 and NP_000537.3.

### E6201 IC50 calculation

Each cell line was plated in triplicate in 200 μL DMEM containing 10% FBS at a density of 3,000 cells per well in 96-well plates. Six hours after cells were seeded, E6201 was added in half log dilutions (3 nM – 10 μM) in triplicate. An equivalent concentration of DMSO was added to untreated wells as a vehicle control. *In vitro* cell proliferation assays were performed using an MTS assay (CellTiter 96 AQueousOne Solution Cell Proliferation Assay, Promega) or SRB (Sulforhodamine B) assay four days after the addition of E6201. IC50 values were calculated using nonlinear regression curve fit with Prism 4 software (GraphPad Software, San Diego, CA). The MTS assay was used for all cell lines except MM329, as this cell line failed to effectively metabolize the MTS reagent; the SRB assay was used in place of the MTS assay in this case. We confirmed in several other melanoma cell lines that both proliferation assays produced comparable IC50 results.

### MTS assay

For the MTS assay, media was removed and 120 μl of media containing 20 μl of MTS (2 mg/mL) and PMS (0.92 mg/mL) was added to each well and incubated for 3 hours at 37°C. Absorbance at 490 nm was measured using a BioTek Synergy HT Multiple Detection microplate reader.

### Sulforhodamine-B (SRB) assay

One of the melanoma cell lines in the current study (MM329) was found not to metabolise the MTS reagent. Therefore, we performed an SRB assay to calculate an IC50 to E6201 for this line. The SRB assay was performed as previously described [51]. Briefly, after drug treatment cells were then fixed with 25 μL of cold trichloroacetic acid (50% w/v) for 60 minutes. Cells were subsequently washed five times with H_2_O and air-dried. Next, cells were stained with 50 μL of 0.04% SRB in 1% acetic acid and incubated at room temperature for 30 minutes. Unbound SRB was removed by washing five times with 1% acetic acid and air-dried. Finally, bound SRB stain was solubilized in 100 μL of 10 mM Tris buffer before taking an optical density measurement at 570 nm using the BioTek microplate reader.

### PI3K and MAPK pathway activation

Cell lines in the panel were plated at a density of 500,000 cells per well on day 0 in a 6-well plate. On day 1, cells were washed twice with PBS, serum-starved in DMEM containing 0.2% FBS and protein lysates were collected 16 hours after serum starvation. 50 μg of total protein were analysed on a 3-8% SDS-PAGE. Phosphorylated Akt and phosphorylated ERK1/2 proteins were probed for with phospho-specific antibodies from Cell Signaling Technology (Beverly, MA). Immunoblots were then stripped and re-probed for total Akt and ERK1/2 (Cell Signaling Technology, Beverly, MA). The ratio of phosphorylated Akt or ERK1/2 to total Akt or ERK1/2 respectively was calculated by densitometry using Image J software and scored as follows: negative 0-15%; + 15-50%, ++ 50-100%; +++ >100% of phosphorylated protein relative to total protein levels. On additional Western blots, PTEN and GAPDH proteins were probed for with antibodies from Cell Signaling Technology (Beverley, MA) and Abcam (Cambridge, MA) respectively.

### Cell cycle analysis

Cells were plated in triplicate in 100-mm^2^ plates. The next day, cells were treated with 200 nM E6201 or 0.01% DMSO (vehicle control). After 48 hours of treatment, cells were fixed in 80% ethanol for 2 hours, washed with ice cold PBS, and then resuspended in 500 μL cell cycle staining buffer (5% FBS, 0.5 mM EDTA, 0.1% Triton X-100, 200 μg/ml propidium iodide, 100 μg/ml RNase A, in PBS). DNA content was evaluated by flow cytometry as an indicator of cell cycle progression. Cell cycle analysis was performed using ModFit software (Verity Software House, Inc. Topsham, ME). The percentage of G1 arrest was calculated as the percent increase in cells in G1 relative to the percent of cells in G1 in DMSO control samples as follows: (% cells in G1 with E6201 - % cells in G1 with DMSO control)/(100-% cells in G1 with DMSO) x 100.

### Cell death analysis by Annexin V staining

Annexin V-FITC staining was used to measure phosphatidylserine exposure on cells undergoing apoptosis according to the manufacturer’s instructions (BioVision, Inc., Mountain View, CA). 2.5 × 10^5^ cells were plated per well in a 6-well plate. Cells were treated with 200 nM E6201 or 0.01% DMSO 24 hours after plating. After 72 hours, floating and attached cells were collected and resuspended in Annexin binding buffer (10 mM Hepes (pH 7.4), 140 mM NaCl, 2.5 mM CaCl_2_). Following the addition of 500 ng/mL Annexin V-FITC and 1 μg/mL propidium iodide (Sigma-Aldrich, St. Louis, MO), cells were analysed for Annexin-positive cells using a CyAn ADP flow cytometer and Summit software, version 4.3 (Dako Cytomation, Carpinteria, CA).

### Cell death analysis by ELISA

*In vitro* determination of cytoplasmic histone-associated DNA fragmentation after E6201 treatment was performed using a 96-well based cell death assay (Cell Death Detection ELISA, Roche). Briefly, cell lines were plated in 200 μL of DMEM plus 10% FBS at a density of 3,000 cells per well on day 0 in two 96-well plates. One plate was used for the ELISA and the other for an SRB assay to estimate total cell number. The next day after plating, 0.01% DMSO vehicle control or 200 nM E6201 was added in triplicate to the corresponding wells of the duplicate 96-well plates. After incubation for 72 hours at 37°C in a humidified incubator, the Cell Death Detection ELISA was performed as per the manufacturer’s instructions. Absorbance was measured at 405 nm using a BioTek microplate reader. The readings from the ELISA were normalized to cell number determined by an SRB assay.

### Murine xenograft melanoma models

Female athymic NU/NU mice were inoculated subcutaneously with 1 × 10^6^ cells from four different *BRAF* mutant (V600E) human melanoma cell lines (MM540, MM604, SKMEL and BL). Once tumours developed to ~100-150 mm^3^, animals were randomized to either vehicle control, or one of three E6201-treated groups, with six mice per group. Vehicle (20% sulfobutylether beta-cyclodextrin in water), or E6201 was administered intravenously via the tail vein at 10, 20 or 40 mg/kg on 3 times per week for 2 weeks. Tumour volume was calculated by calliper measurement (mm) using the following formula: (l × w^2^)/2 = mm^3^ where “l” and “w” refer to the larger and smaller dimensions obtained at each measurement. All animal studies were approved by the Eisai Animal Care and Use Committee.

### E6201 and LY294002 combination study

Synergy between E6201 and LY294002 was evaluating using a non-fixed ratio method, such that fixed concentrations of LY294002 (1 μM, 5 μM, 10 μM, 20 μM, 30 μM) were added with increasing concentrations of E6201 (3 nM to 3 μM). Briefly, each cell line was plated in 200 μL DMEM containing 10% FBS and L-glutamine at a density of 3,000 cells per well on day 0 in 96-well plates. On day 1, 25 μL of 10X concentrated serial half-log dilutions of E6201 were added in triplicate for final concentrations ranging from 3 μM to 3 nM. After E6201 was added to each plate, 25 μL of 10X concentrated LY294002 was added in triplicate for final concentrations of 30 μM, 20 μM, 10 μM, 5 μM, or 1 μM. Each plate contained control wells for vehicle (0.33% DMSO) alone, LY294002 alone, and E6201 alone, in triplicate. For single agent IC50 generation, E6201 was added in half-log serial dilution from 10 μM to 3 nM and LY294002 from 50 μM to 1 μM. After the addition of E6201 and LY294002, cells were incubated for 72 hours at 37°C and the SRB assay was then performed as described above.

### Statistics

Data from proliferation assays were imported into Excel and processed to subtract the MTS or SRB background from each data point. Each data point was then normalized to the average absorbance of the DMSO vehicle control wells on its same 96-well plate. These ‘percent DMSO control’ data were used to graph the concentration response curves and to calculate the IC50 values for each drug alone using non-linear regression analysis with Prism software (GraphPad Software, San Diego, CA). Significant association was determined using the Fisher’s Exact Test.

Synergy was analysed by the Chou and Talalay combination index method using CalcuSyn software. The ‘percent DMSO control’ data was averaged for each combination and converted to effect values using the following equation: [effect = 1 – (‘Average Percent of DMSO control’)/100] prior to being imported into CalcuSyn for calculation of the combination index. Any effect values that were less than 0 (i.e. the drug treated wells produced absorbance values greater than those seen in wells treated with DMSO alone) were set to 0.001 for analysis. As these values were artificially set at 0.001, any combination index values that were generated from drug combinations that were set to 0.001 were excluded from graphs of the combination index values. A combination index value of 1 indicates additivity, values < 1 indicate synergism, and values > 1 indicate antagonism.

## Abbreviations

Akt: Protein kinase B; CI: Combination index; DMSO: Dimethyl sulfoxide; ELISA: Enzyme-linked immunosorbent assay; ERK: Extracellular signal-regulated kinase; IGF-1R: Insulin-like growth factor 1 receptor; MAPK: Mitogen-activated protein kinase; MEK: Mitogen-activated protein kinase/extracellular signal-regulated kinase kinase; mTOR: Mammalian target of rapamycin; PI3K: Phosphoinositide 3-kinase; *PTEN*: Phosphatase and tensin homolog; Ras: Rat sarcoma; SRB: Sulforhodamine B.

## Competing interests

This study was supported in part by a grant and drug from Eisai Inc., Andover, Massachusetts, USA. JWu and KN are both employees of Eisai Inc. JWa is an employee of H3 Biomedicine Inc. H3 Biomedicine Inc. is a subsidiary of Eisai Inc.

## Authors’ contributions

SB carried out many of the *in vitro* experiments, including proliferation assays, cell cycle and cell death analyses, E6201/LY294002 combination studies and Western blots. SB also helped to draft the manuscript. DL performed some of the Western blots and helped to draft the manuscript. CW performed proliferation assays, cell cycle and cell death analyses, E6201/LY294002 and E6201/Rapamycin combination studies and Western blots. AW performed some of the Western blots and helped to draft the manuscript. JWu, JWa and KN carried out the xenograft experiments, participated in the study’s design and helped to draft the manuscript. PP conceived of the study, and participated in its design and coordination and helped to draft the manuscript. All authors read and approved the final manuscript.

## Supplementary Material

Additional file 1 ** Figure S1.** Efficacy of MEK1/2 inhibition with E6201. Western blots demonstrating phosphorylated ERK1/2 levels in our panel of melanoma cell lines following treatment with either vehicle (0.05% DMSO) or 500 nM E6201. Briefly, 500,000 cells from each cell line were plated in duplicate in a 6-well plate on day 0. The next day cells were washed twice with PBS and serum-starved in DMEM containing 0.2% FBS. Sixteen hours after serum starvation, cells were treated with either 0.05% DMSO or 500 nM E6201. After 6 hours of treatment protein lysates were collected. 30 μg of total protein were analysed on a 12% SDS-PAGE gel. Phosphorylated ERK1/2 protein was probed for with a phospho-specific antibody from Cell Signaling Technology (Beverly, MA). Immunoblots were then stripped and re-probed for total ERK1/2 (Cell Signaling Technology, Beverly, MA) and tubulin (Sigma Aldrich, St Louis, MO). Click here for file

Additional file 2 ** Figure S2.** Efficacy of PI3K inhibition with LY294002. Western blots demonstrating phosphorylated AKT (serine 473) levels in UACC647, UACC558, UACC903, MM622, WM35 and MM540 cell lines following treatment with either vehicle (0.15% DMSO) or 20 μM LY294002. Briefly, 500,000 cells from each cell line were plated in duplicate in a 6-well plate on day 0. The next day cells were washed twice with PBS and serum-starved in DMEM containing 0.2% FBS. Sixteen hours after serum starvation, cells were treated with either 0.15% DMSO or 20 μM LY294002. After 6 hours of treatment, protein lysates were collected. 30 μg of total protein were analysed on a 12% SDS-PAGE gel. Phosphorylated AKT protein was probed for with a phospho-specific antibody from Cell Signaling Technology (Beverly, MA). Immunoblots were then stripped and re-probed for total AKT (Cell Signaling Technology, Beverly, MA) and GAPDH (Abcam, Cambridge, MA). Click here for file

Additional file 3 ** Figure S3.** LY294002 Single Agent Concentration Response Curves. Concentration response curves of UACC647, UACC558, UACC903, MM622, WM35 and MM540 melanoma cell lines to the PI3K inhibitor LY294002. The IC50 of LY294002 for each cell line is provided in the legend. Click here for file

Additional file 4 ** Figure S4.** Concentration response curves for E6201 and LY294002 combinations normalized to DMSO. Concentration response curves of UACC647, UACC558, UACC903, MM622, WM35 and MM540 melanoma cell lines to increasing concentrations of E6201 (3 nM to 3 μM) in combination with LY294002 (1 μM, 5 μM, 10 μM, 20 μM and 30 μM) treatment. Click here for file
