# Supplementary figures and images for: Sensitivity to the MEK inhibitor E6201 in melanoma cells is associated with mutant BRAF and wildtype PTEN status
Source: Mol Cancer. 2012 Oct 5;11:75. doi: 10.1186/1476-4598-11-75 (PMC3554420; doi:10.1186/1476-4598-11-75)

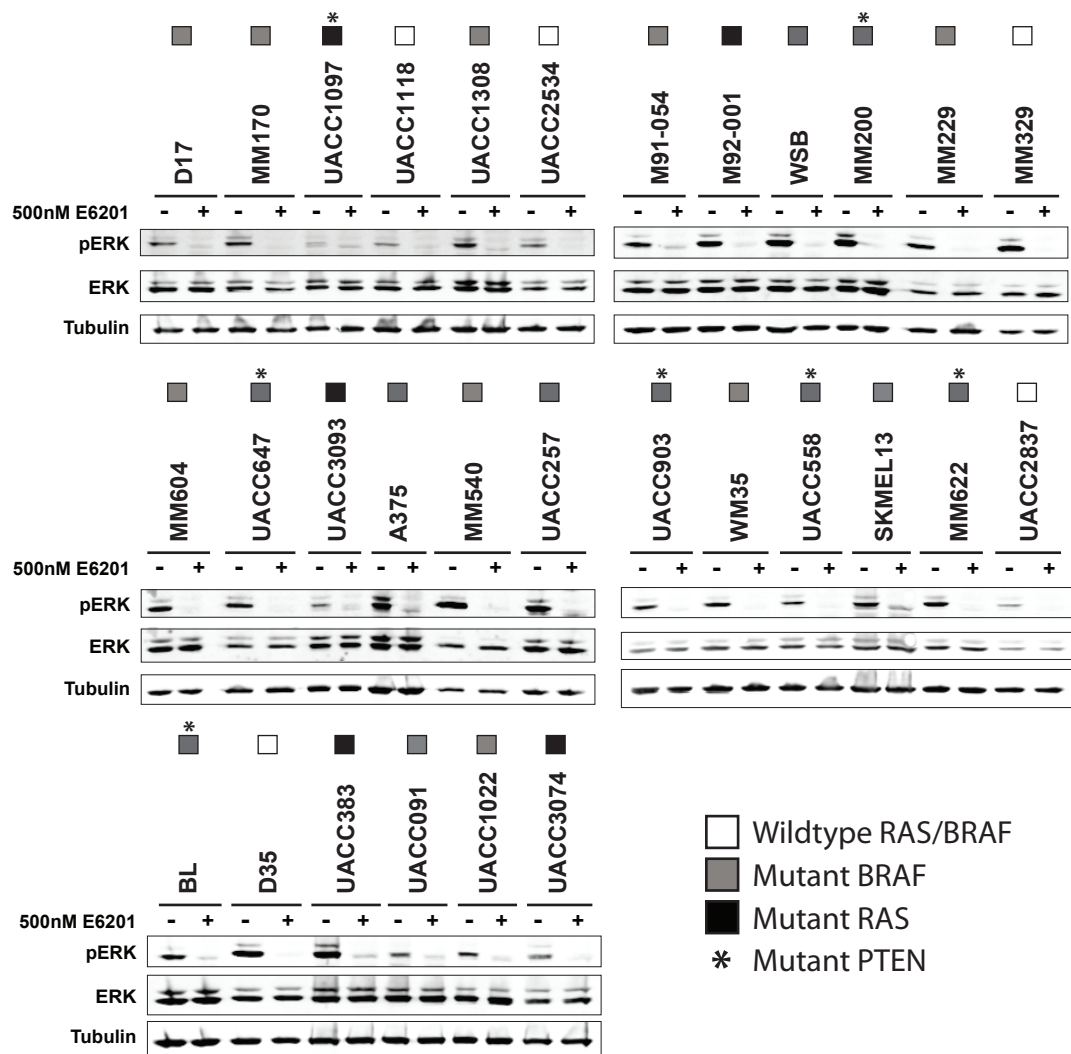

Supplement: Additional file 1 — Figure S1. Efficacy of MEK1/2 inhibition with E6201. Western blots demonstrating phosphorylated ERK1/2 levels in our panel of melanoma cell lines following treatment with either vehicle (0.05% DMSO) or 500 nM E6201. Briefly, 500,000 cells from each cell line were plated in duplicate in a 6-well plate on day 0. The next day cells were washed twice with PBS and serum-starved in DMEM containing 0.2% FBS. Sixteen hours after serum starvation, cells were treated with either 0.05% DMSO or 500 nM E6201. After 6 hours of treatment protein lysates were collected. 30 μg of total protein were analysed on a 12% SDS-PAGE gel. Phosphorylated ERK1/2 protein was probed for with a phospho-specific antibody from Cell Signaling Technology (Beverly, MA). Immunoblots were then stripped and re-probed for total ERK1/2 (Cell Signaling Technology, Beverly, MA) and tubulin (Sigma Aldrich, St Louis, MO). [file 1476-4598-11-75-S1.pdf]

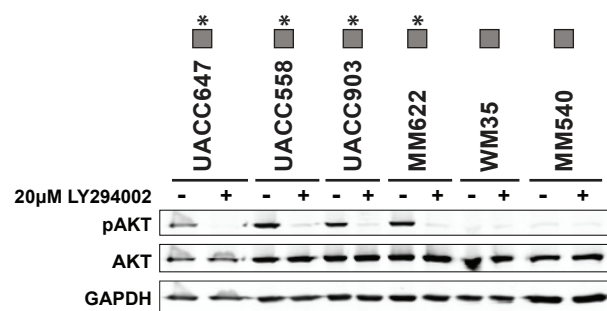

Mutant BRAF  
 \* Mutant PTEN

Supplement: Additional file 2 — Figure S2. Efficacy of PI3K inhibition with LY294002. Western blots demonstrating phosphorylated AKT (serine 473) levels in UACC647, UACC558, UACC903, MM622, WM35 and MM540 cell lines following treatment with either vehicle (0.15% DMSO) or 20 μM LY294002. Briefly, 500,000 cells from each cell line were plated in duplicate in a 6-well plate on day 0. The next day cells were washed twice with PBS and serum-starved in DMEM containing 0.2% FBS. Sixteen hours after serum starvation, cells were treated with either 0.15% DMSO or 20 μM LY294002. After 6 hours of treatment, protein lysates were collected. 30 μg of total protein were analysed on a 12% SDS-PAGE gel. Phosphorylated AKT protein was probed for with a phospho-specific antibody from Cell Signaling Technology (Beverly, MA). Immunoblots were then stripped and re-probed for total AKT (Cell Signaling Technology, Beverly, MA) and GAPDH (Abcam, Cambridge, MA). [file 1476-4598-11-75-S2.pdf]

UACC647 and UACC558

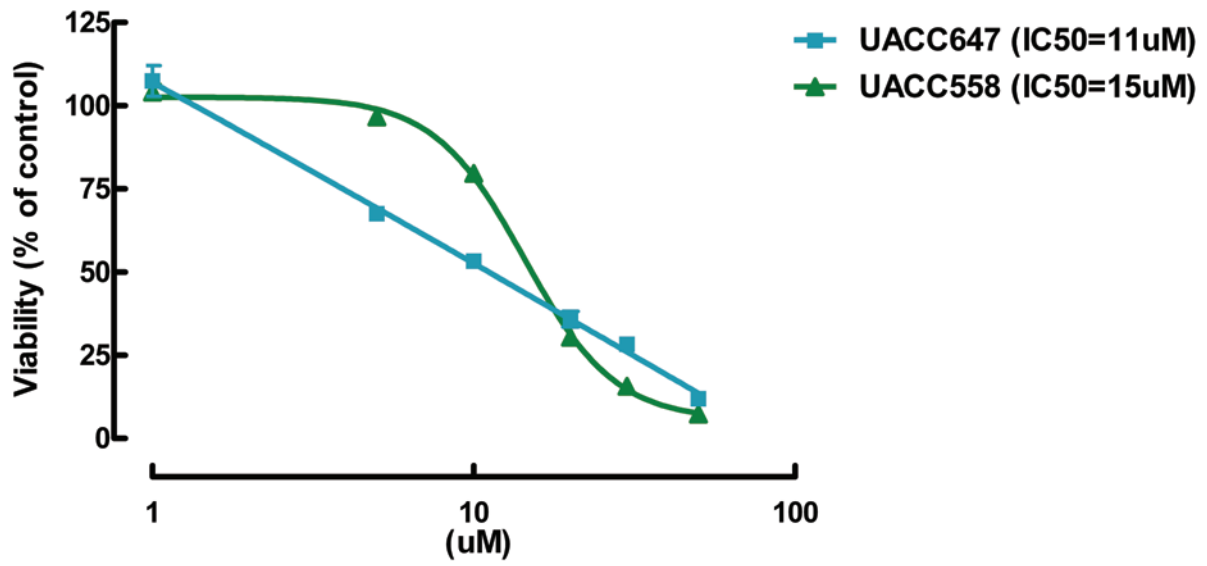

UACC903 and MM622

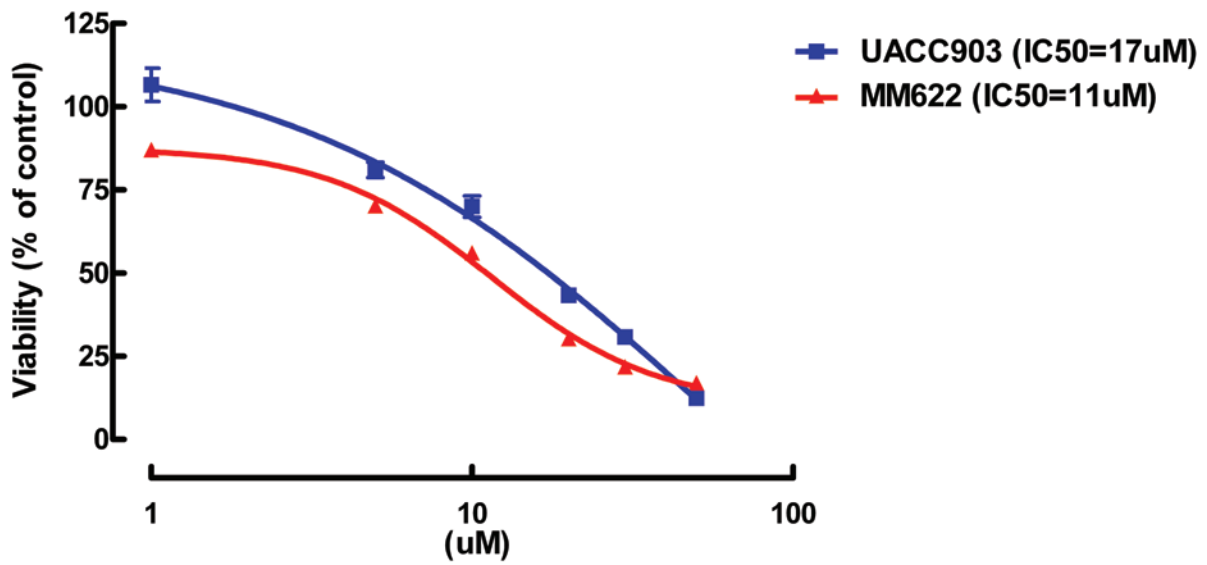

WM35 and MM540

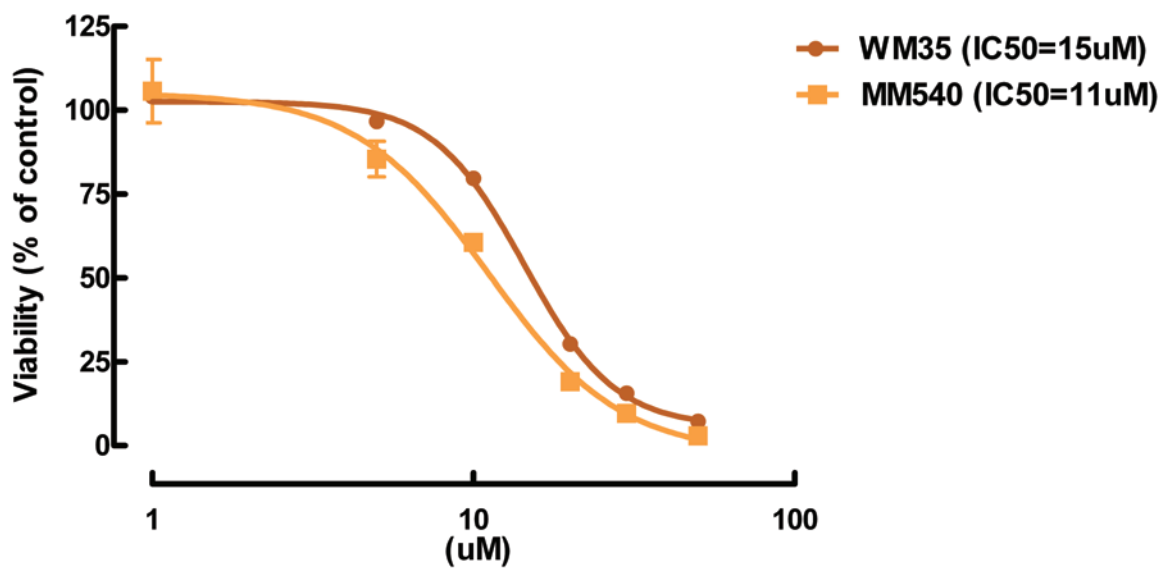

Supplement: Additional file 3 — Figure S3. LY294002 Single Agent Concentration Response Curves. Concentration response curves of UACC647, UACC558, UACC903, MM622, WM35 and MM540 melanoma cell lines to the PI3K inhibitor LY294002. The IC50 of LY294002 for each cell line is provided in the legend. [file 1476-4598-11-75-S3.pdf]

UACC647

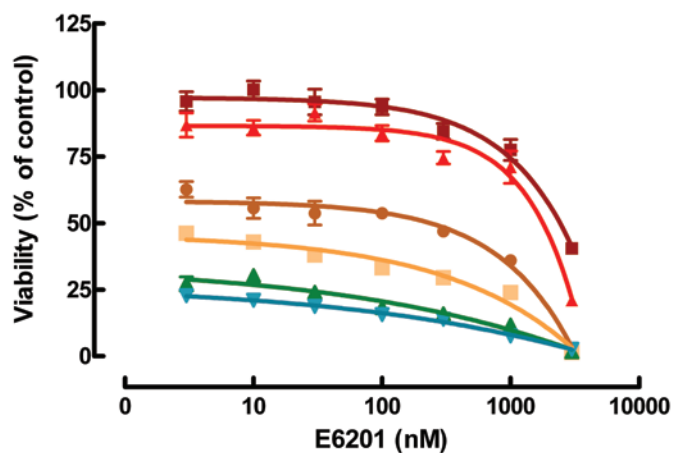

UACC558

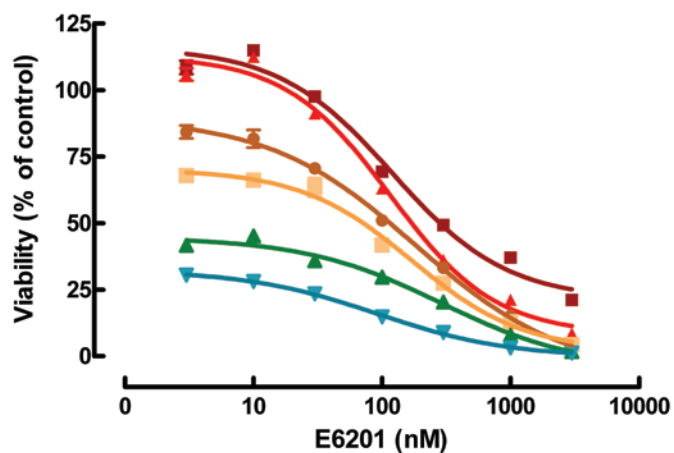

UACC903

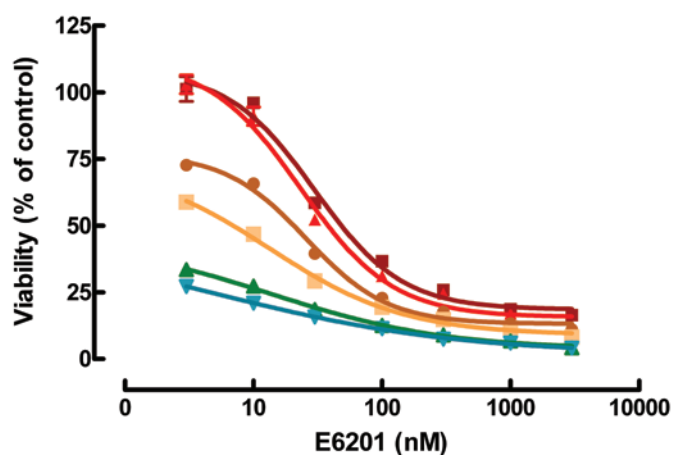

MM622

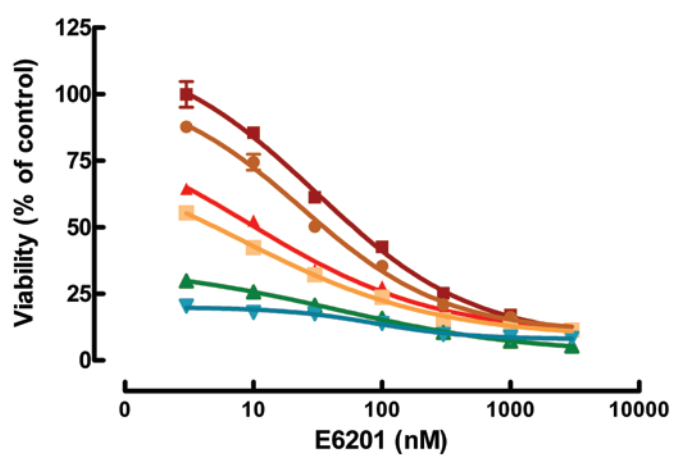

WM35

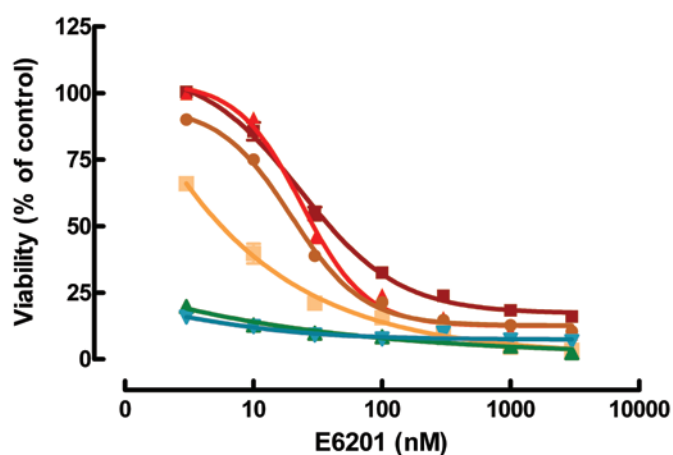

MM540

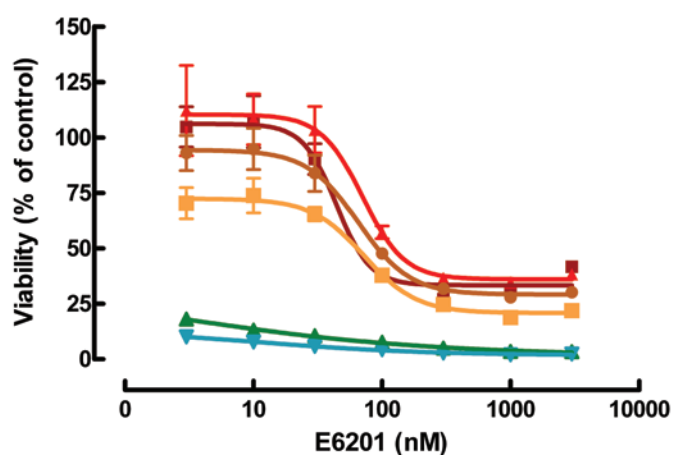

- E6201
- ▲ E6201 + 1uM LY294002
- E6201 + 5uM LY294002
- E6201 + 10uM LY294002
- ▲ E6201 + 20uM LY294002
- ▼ E6201 + 30uM LY294002

Supplement: Additional file 4 — Figure S4. Concentration response curves for E6201 and LY294002 combinations normalized to DMSO. Concentration response curves of UACC647, UACC558, UACC903, MM622, WM35 and MM540 melanoma cell lines to increasing concentrations of E6201 (3 nM to 3 μM) in combination with LY294002 (1 μM, 5 μM, 10 μM, 20 μM and 30 μM) treatment. [file 1476-4598-11-75-S4.pdf]
